# Development of a shortwave infrared sinuscope for the detection of cerebrospinal fluid leaks

**DOI:** 10.1117/1.JBO.28.9.094803

**Published:** 2023-05-12

**Authors:** Tjadina-W. Klein, Stella Yang, Mahbuba A. Tusty, Jayakar V. Nayak, Michael T. Chang, Oliver T. Bruns, Thomas S. Bischof, Tulio A. Valdez

**Affiliations:** a German Cancer Research Center (DKFZ), Heidelberg, Germany; b National Center for Tumor Diseases (NCT/UCC), Dresden, Germany; German Cancer Research Center (DKFZ), Heidelberg, Germany; Medizinische Fakultät and University Hospital Carl Gustav Carus, Technische Universität Dresden, Dresden, Germany; Helmholtz-Zentrum Dresden-Rossendorf (HZDR), Dresden, Germany; c Helmholtz Zentrum München, Helmholtz Pioneer Campus, Neuherberg, Germany; d Ludwig-Maximilians-Universität München, Medizinische Fakultät, Munich, Germany; e Stanford University, Department of Otolaryngology, Head and Neck Surgery, Palo Alto, California, United States; f Technische Universität München, Department of Medicine, Munich, Germany

**Keywords:** skull base surgery, shortwave infrared, sinuscopy, endoscopy, cerebrospinal fluid leak, NIR-II

## Abstract

**Significance:**

Cerebrospinal fluid (CSF) rhinorrhea (leakage of brain fluid from the nose) can be difficult to identify and currently requires invasive procedures, such as intrathecal fluorescein, which requires a lumbar drain placement. Fluorescein is also known to have rare but significant side effects including seizures and death. As the number of endonasal skull base cases increases, the number of CSF leaks has also increased for which an alternative diagnostic method would be highly advantageous to patients.

**Aim:**

We aim to develop an instrument to identify CSF leaks based on water absorption in the shortwave infrared (SWIR) without the need of intrathecal contrast agents. This device needed to be adapted to the anatomy of the human nasal cavity while maintaining low weight and ergonomic characteristics of current surgical instruments.

**Approach:**

Absorption spectra of CSF and artificial CSF were obtained to characterize the absorption peaks that could be targeted with SWIR light. Different illumination systems were tested and refined prior to adapting them into a portable endoscope for testing in 3D-printed models and cadavers for feasibility.

**Results:**

We identified CSF to have an identical absorption profile as water. In our testing, a narrowband laser source at 1480 nm proved superior to using a broad 1450 nm LED. Using a SWIR enabling endoscope set up, we tested the ability to detect artificial CSF in a cadaver model.

**Conclusions:**

An endoscopic system based on SWIR narrowband imaging can provide an alternative in the future to invasive methods of CSF leak detection.

## 1 Introduction

The diagnosis of cerebrospinal fluid (CSF) leaks can be challenging, and a missed or delayed diagnosis can lead to potentially life-threatening complications, such as meningitis.^1^^–^^3^ With recent advancements in endoscopic skull base surgery, the incidence of CSF leaks continues to rise, with as high as 30.1% of cases having an intraoperative CSF leak, making iatrogenic causes the leading etiology.^4^ In this setting, and particularly in the acute postoperative period, CSF rhinorrhea can be difficult to distinguish clinically from blood and mucus drainage. Other scenarios where a CSF leak may be difficult to discern and locate are in the presence of craniofacial trauma and spontaneous CSF leaks.^5^

Existing options for CSF leak diagnosis, such as intrathecal fluorescein or Beta2 transferrin, have limitations in immediacy of results and introduce potential morbidity.^6^ One possible method to improve diagnostic ability for CSF leak involves shortwave infrared (SWIR) radiation technology. We refer to SWIR as the electromagnetic spectrum ranging from 1000 to 2000 nm which is not visible to the human eye and conventional silicon-based cameras.^7^ Compared to traditional visible and near-infrared (NIR) fluorescence imaging, SWIR is capable of providing greater contrast, sensitivity, and penetration depths. Moreover, tissue components, such as water, lipids, and collagen, have more prominent SWIR absorption features than corresponding features seen in the visible and NIR.^8^^,^^9^ Still so, SWIR has not yet been readily adapted to clinical settings or diagnostics. This is partly because suitable detectors, until recently, have been either inaccessible or cost prohibitive. Recent studies have highlighted the potential of SWIR technology in clinical diagnostics.^10^ Fluid in the middle ear, for example, shows strong light absorption between 1400 and 1550 nm. As a result, straightforward middle ear fluid detection in a model using a SWIR otoscope is possible and can be used to diagnose otitis media with greater accuracy than is currently possible with a standard otoscope.^11^^,^^12^ SWIR technology can also be used to improve the characterization of allergic contact dermatitis using the different absorption properties of different SWIR channels.^13^

In this paper, we describe the development of an endoscopic surgical system for assessment of CSF leaks of the skull base, based on SWIR imaging. To begin, we describe the physical principles behind the contrast mechanism and estimate potential performance using *in vitro* characterization. We then develop a benchtop imaging system and perform proof-of-principle experiments. After testing the initial endoscope design, we identify points that can be optimized. We show that the detection quality can significantly be improved when moving to a narrower illumination band and adding polarizers to the design. Using a porcine nasal cavity, we show that the improved design is capable of detecting very small amounts of fluid in a nasal cavity.

## 2 Methods

### 2.1 System/Design

**Fig. 1:**
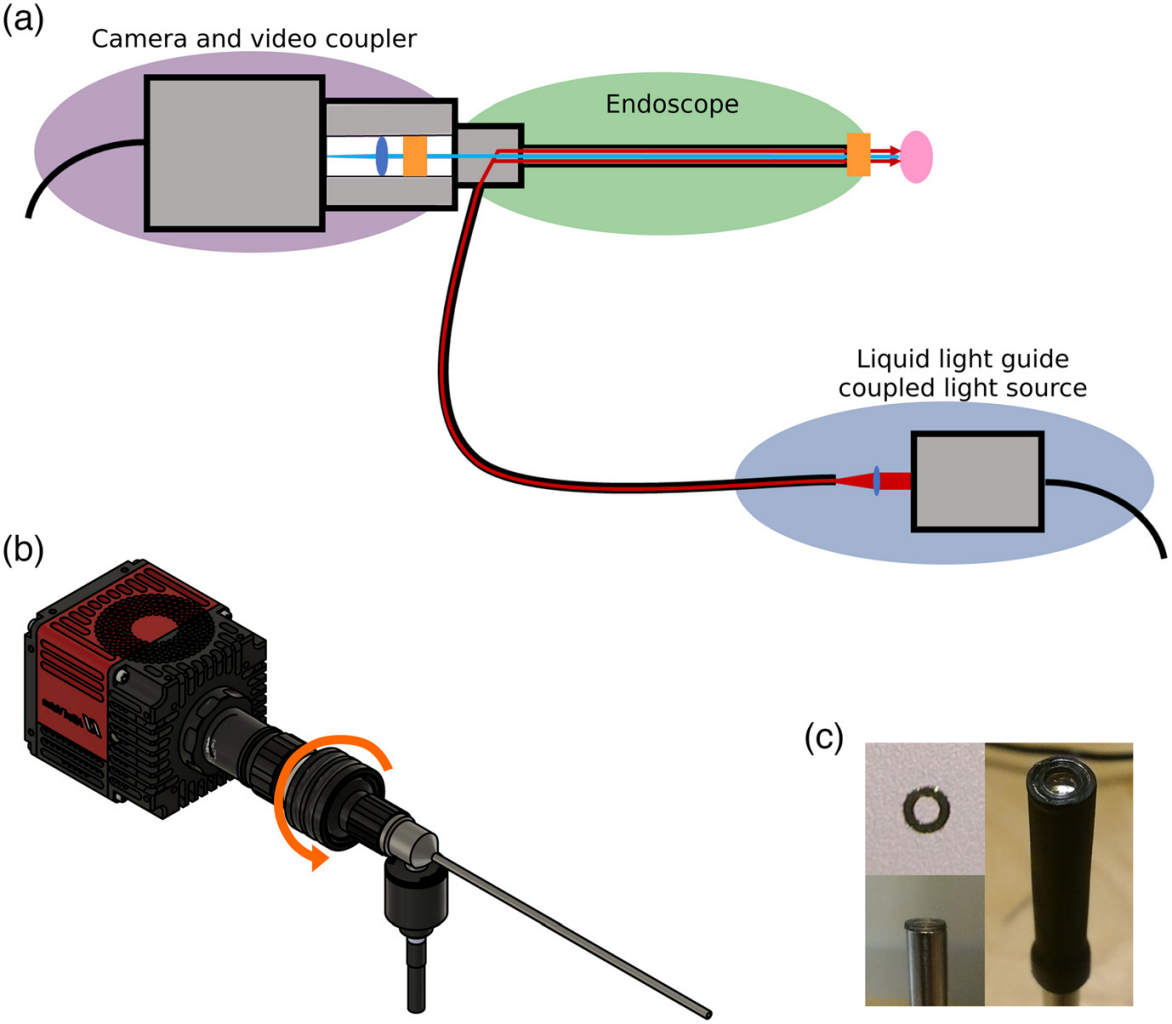
Design of the SWIR endoscope. (a) Schematic of the SWIR endoscope, consisting of an InGaAs imager, a video coupler containing a lens and polarizer, a SWIR-optimized rigid endoscope with a second polarizer, and a liquid light guide coupled light source. The blue ellipses and orange rectangles indicate the positions of the lenses and polarizers. The red lines show the path of the illumination beam, whereas the blue line represents the light reflected off the sample and collected by the camera. (b) CAD design of the SWIR endoscope. The orange arrow shows the part of the setup that can be rotated to align the two polarizers. (c) Images of the polarizers used for the endoscope setup: the custom cut polarizer, top view; positioning of the polarizer on the tip of the endoscope; the polarizer covering only the fiber ring and not the lens, held in position by a sleeve (top to bottom and left to right).

We based our SWIR endoscope on the design of a regular rigid white light surgical endoscope. A Hawkeye Pro Slim endoscope (Gradient Lens Corporation) was optimized for the wavelength range 900 to 1700 nm using a custom coating. An off the shelf video coupler (Gradient Lens Corporation VC-35) was modified by exchanging the original lens for a SWIR compatible lens. The SWIR camera was attached to the video coupler using a c-mount extension tube. Two different SWIR cameras were used for this project: the Goldeye G-032 Cool TEC2 (Allied Vision) and the Owl 640 Mini VIS-SWIR (Raptor Photonics). Two polarizers were incorporated into the endoscope (see schematic in Fig. 1). One was positioned at the tip of the endoscope (#12-473, Edmund Optics), the other was positioned in the video coupler in front of the lens (LPNIR050, Thorlabs). Using a laser cutter, the polarizer at the tip of the endoscope was custom cut to fit exactly over the fiber ring of the endoscope without covering the lens of the endoscope. The polarizers were oriented orthogonal to each other to minimize direct reflections. The positioning of the polarizers can be seen in Fig. 1.

The illumination for the endoscope consists of a narrowband 1480-nm laser (Thorlabs L1480G1), which is coupled into the endoscope using a liquid light guide (Thorlabs LLG3-4Z). A lens (Thorlabs C060TMD-C) was used to focus the laser into the liquid light guide and a computer fan with a blade broken off was used to reduce laser speckle.

### 2.2 Comparison of White Light SWIR Performance for Visualizing CSF

Transmission spectra were taken of human CSF, artificial CSF (aCSF) (EcoCyte Bioscience), and water. aCSF has an electrolyte concentration closely matching CSF with final ion concentrations of NaCl 125 mM, KCl 3.0 mM, ${\mathrm{CaCl}}_{2}$ 2.5 mM, ${\mathrm{MgSO}}_{4}$ 1.3 mM, and ${\mathrm{NaH}}_{2}{\mathrm{PO}}_{4}$ 1.25 mM along with high-purity water. 700 microliter volumes of either aCSF or water were transferred to 2-mm pathlength quartz cuvettes (Thorlabs CV10Q7F) to measure attenuation spectra using Lambda 1050+ spectrophotometer (Perkin Elmer). The human CSF spectrum was taken, along with a second water spectrum for comparison, at our clinical site using a Cary 6000i (Agilent).

To investigate the potential of the SWIR absorption band seen in the spectroscopic data for fluid detection in imaging data, we imaged three Eppendorf tubes: one partially filled with water, one partially filled with aCSF, and one empty. They were placed next to each other on a white background and illuminated and imaged from above. The white light image was taken using a white light LED (Thorlabs MNWHL4), an IDS camera (UI-3880SE-C-HQ), and a Navitar lens (Navitar HR F1.4/8 mm). The SWIR image was taken using the Goldeye G-032 Cool TEC2 camera, a Navitar SWIR-35 lens, and a broadband 1450-nm LED (Thorlabs M1450L3).

The SWIR setup used for imaging the water on the chicken skin was identical to that used to image the Eppendorf tubes. For the white light imaging of the chicken skin, an IDS camera (UI-3880SE-C-HQ) was used, in combination with a Navitar lens (Navitar HR F1.4/8 mm) and a white light LED for illumination (Thorlabs MNWHL4).

### 2.3 Cadaver Study

To evaluate ease of use and proof of principle for visualization of CSF on the skull base, we performed a cadaver study. A trephine was performed through an infrabrow incision to access the frontal sinus and a communication was achieved with the intracranial compartment by puncturing the posterior table of the frontal sinus. A catheter was then inserted into the defect. An intranasal injury to the skull base was performed allowing communication for the artificial CSF. The aCSF pumped from the frontal sinus could be assessed from the nasal cavity with our endoscope. For imaging, the SWIR endoscope was used, with a 1450-nm LED light source, the Owl 640 Mini, and no polarizers.

### 2.4 Use of a Narrowband Illumination Improves CSF Contrast

The initial test to assess the effect narrowing the illumination band has on our imaging data was done using the macrosetup described above. As a sample, we used a Petri dish filled with a small amount of water and placed above a test target. To compare relatively broadband to narrowband illumination, we performed imaging using a 1450-nm LED (Thorlabs M1450L3) to the same LED with an excitation filter (1480 nm center, 12 nm width; Thorlabs FB1480-12). The depth of the water was roughly 1 mm (slightly less in the middle of the Petri dish).

Using the macrosetup and the 1450-nm LED (Thorlabs M1450L3) without a filter versus the same 1450-nm LED with the excitation filter (1480 nm center, 12 nm width; Thorlabs FB1480-12), we also imaged a piece of chicken skin, both without water present and with a droplet of water running across it.

For the assessment in the endoscopic setup, we again used a partially water filled Petri dish above a test target. This time the Petri dish was slightly angled to create a gradient of water thickness. A very small amount of dish soap was added to the water to reduce the surface tension and create a thinner film of water. The maximum thickness of water in the images is around 1 mm, and the thinnest part is ≪1  mm. These images were taken without the use of polarizers. For the broadband illumination, we used a 1450-nm LED (Thorlabs M1450L3), for the narrowband illumination we used a 1480-nm laser (Thorlabs L1480G1). For the macro and the endoscopic images, the Goldeye G-032 was used.

### 2.5 Reducing Reflections Through the Use of Polarizers

One polarizer was positioned inside the video coupler (between endoscope and camera), and a second polarizer was attached to the tip of the endoscope. The polarizer at the tip of the endoscope was custom cut using a laser cutter to only cover the fiber ring of the endoscope and not the lens. The final dimensions of the polarizers were 4.3 mm (outer diameter) and 2.5 mm (inner diameter). The polarizers were oriented orthogonal to each other to bring the reflections to a minimum. Images were taken using the endoscopic setup (1480-nm laser, Goldeye G-032 camera) (first without polarizers, then with polarizers added in), and the sample was a piece of chicken skin. Water was dripped onto the chicken skin during imaging.

### 2.6 Comparison of Calculated and Measured Values

A piece of nasal turbinate was taken from a porcine nasal cavity and a reflection spectrum was measured using a Lambda 1050+ spectrophotometer (Perkin Elmer). A transmission spectrum of artificial CSF (aCSF) (EcoCyte Bioscience) was also taken using the same instrument. The resulting data were used to calculate the contrast we would expect to see between the porcine nasal tissue and the aCSF if using an illumination source centered on the absorption peak of aCSF.

To be able to compare the calculated value with an actual measurement, we imaged a small pool of aCSF inside a porcine nasal cavity. We used our endoscope setup (1480-nm laser illumination, Goldeye G-032 camera), and the pool of aCSF was about 2 mm deep. The resulting image was used to calculate the actual contrast between the porcine nasal tissue and the aCSF.

### 2.7 Imaging the Nasal Cavity

To assess our system in a realistic nasal environment, we imaged a porcine nose using the improved endoscope system. The endoscope system consisted of the narrowband laser light source and the polarizers, as described above (Sec. 2.1), and the Goldeye G-032 camera was used. Our sample was a food grade half pig head. The pig head had been cut in half along the sagittal plane, making it possible to drip water directly into the nasal canal. The endoscope was inserted into the nasal canal and water was dripped into the nasal canal so that it drained toward the endoscope and the tip of the nose.

To compare the SWIR images to white light images, we also imaged the porcine nasal cavity using a white light endoscope (Schindler Endoskope). The camera was the same as previously used for white light imaging (IDS UI-3880SE-C-HQ), and we used a broadband white light source for illumination (Thorlabs OSL2).

## 3 Results

### 3.1 Comparison of White Light SWIR Performance for Visualizing CSF

**Fig. 2:**
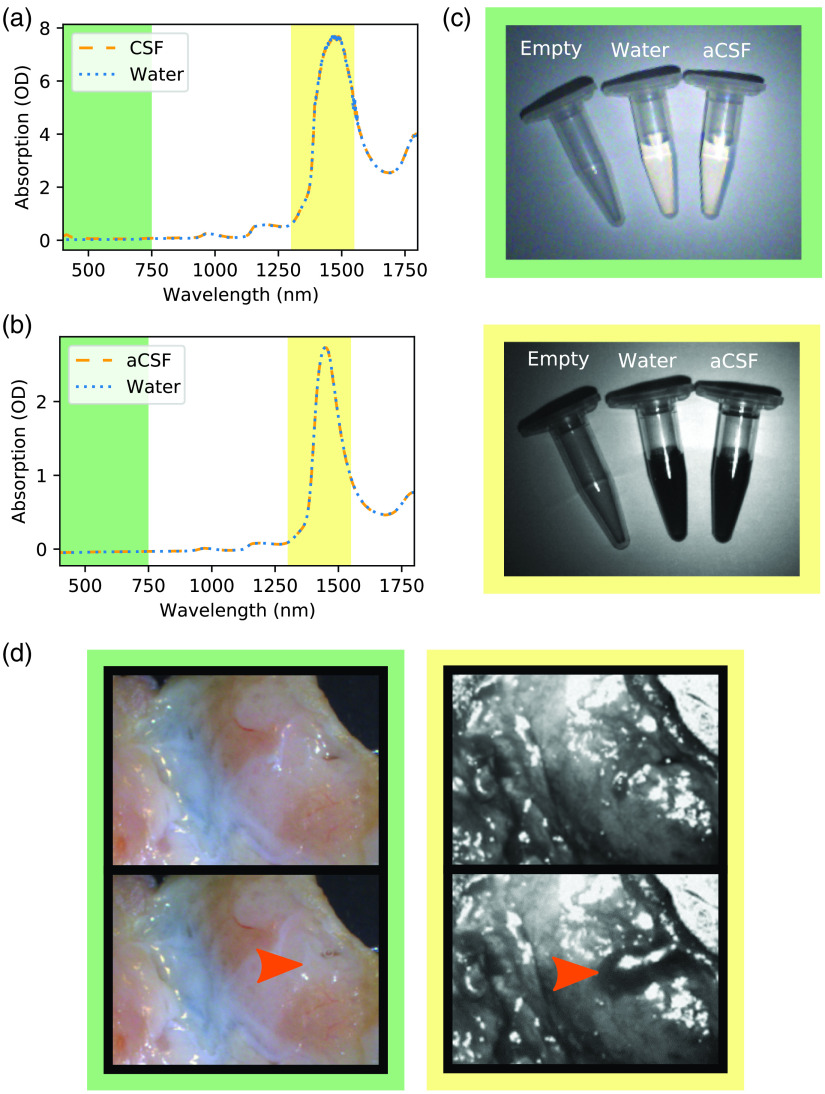
Optical properties of water, aCSF, and human CSF: comparison of the absorption spectra of (a) water and human CSF and (b) water and aCSF. In both (a) and (b), there are no significant differences in the spectra in the range of 400 to 1800 nm. The differences in OD between human CSF and aCSF are due to a difference in path length for the human CSF spectrum and the aCSF spectrum and the use of different instruments. A water spectrum was taken along with each of the (a) CSF samples to show that the difference in OD is due to different path lengths/instruments and not due to different absorption properties of human CSF and aCSF. (c) Eppendorf tubes from left to right: empty, water, and aCSF. Both water and aCSF show strong absorption at ∼1450  nm, which causes them to appear black, whereas they are translucent in the visible wavelength range. (d) Comparison of tissue without and with the presence in water. Although in the visible spectrum, the presence of water cannot clearly be defined, when using the broadband 1450-nm SWIR imaging, the water produces a much clear contrast to the surrounding tissue and is clearly visible as a dark area. The orange arrows indicate the area where water is present. The background colors correspond to the highlighted wavelength ranges in the spectra.

To test our hypothesis that the absorption properties of CSF are similar to water and to predict whether a SWIR endoscope could be beneficial for CSF detection, we investigated the optical properties of CSF. The spectroscopic analysis confirmed that the properties of human CSF and aCSF match those of water for the range of 400 to 1800 nm. The suspected absorption band of human CSF and aCSF at around 1450 nm was clearly visible in the spectroscopic data [Fig. 2(a) and 2(b)].

The imaging of the Eppendorf tubes confirmed that while being transparent in the visible there was strong attenuation when moving the 1450-nm SWIR imaging window, for both water and aCSF; whereas in the visible the fluid in the Eppendorf tubes is translucent, it appears dark and opaque in the SWIR and has a stark contrast to the air-filled Eppendorf tube and the background [Fig. 2(c)]. Having confirmed that the optical properties of water, human CSF, and aCSF in the SWIR are near identical, we will use water for all experiments from this point on, due to water being readily available.

Since skin and other biological tissues have a high water content, we were expecting a reduction in contrast between water and background when moving to imaging the water on top of tissue. We tested whether moving to the SWIR would give us an advantage in the detection of water on top of tissue compared to white light imaging using a piece of chicken skin (food grade; inner side of skin facing upward/toward camera). Using a piece of plastic tubing inserted through the chicken skin, water could be pooled onto the surface of the chicken skin. The resulting images showed that in the visible there is no significant difference to be seen between chicken skin with and without water. In the SWIR, however, the water droplet on top of the chicken skin can clearly be depicted against the surrounding skin [Fig. 2(d)].

### 3.2 Cadaver Study

**Fig. 3:**
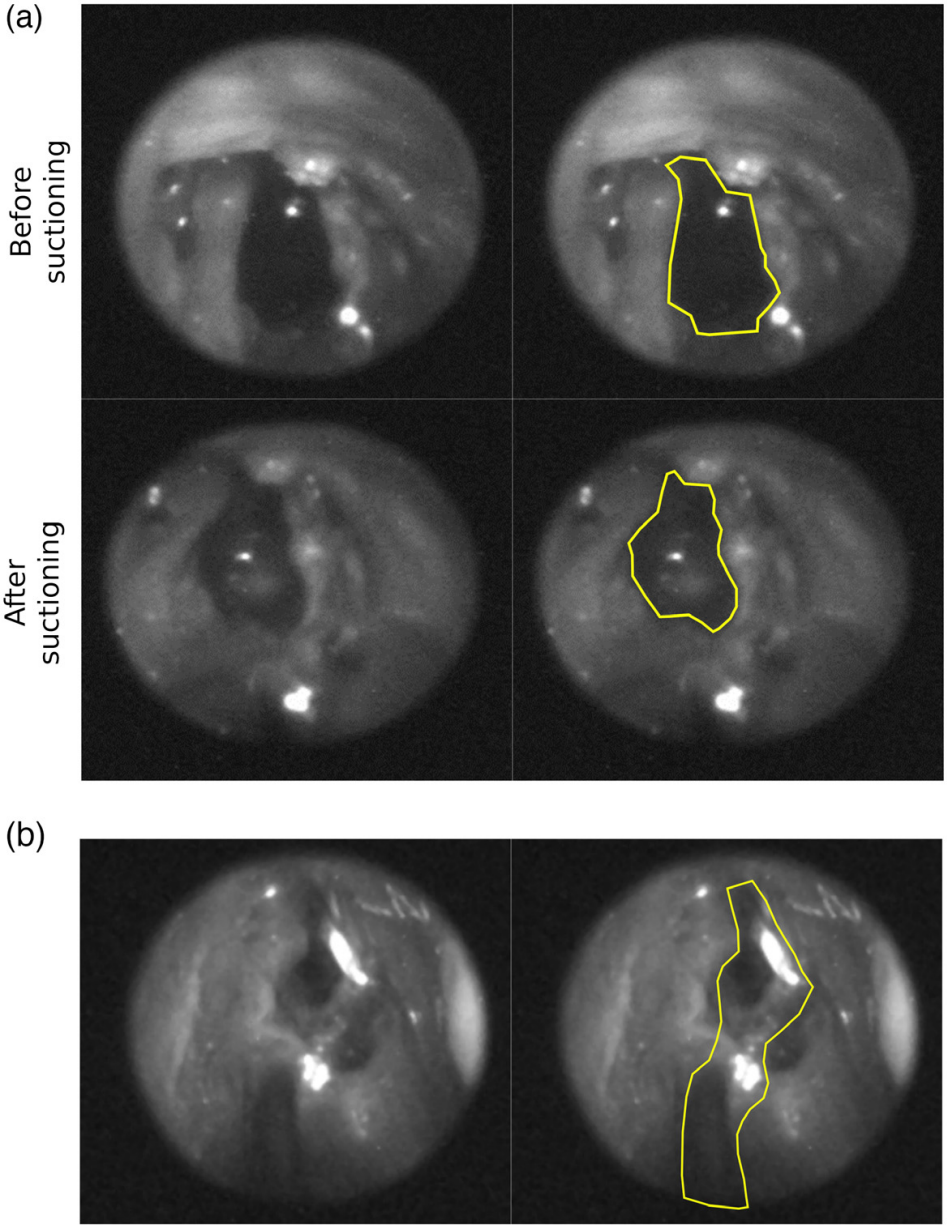
Imaging the skull base of a cadaver using the SWIR endoscope. (a) The endoscopic SWIR images show that larger accumulations of aCSF can be detected due to the increased amount of absorption. The yellow outline shows the cavity in which aCSF accumulated, the left and right SWIR images are otherwise identical. (b) Detection of thin films of aCSF is difficult as there is not enough contrast between the thin films and the surrounding and underlying tissue. In the right image, we have highlighted the area where water is present. For comparison, the left image is identical to the right, other than the water not being outlined. This shows that accurate detection of thin films of aCSF was not possible with the endoscope design used for the cadaver study, which incorporated a 1450-nm LED without an excitation filter.

The cadaver imaging confirmed that we can detect an accumulation of artificial CSF inside the human nasal cavity using the SWIR endoscope. The SWIR endoscope system is also able to show anatomical landmarks comparable to scopes currently used in sinus surgery. Detecting thin films of artificial CSF proved difficult, highlighting the need to make modifications to the system to be able to detect smaller amounts of liquid (Fig. 3).

### 3.3 Use of a Narrowband SWIR Illumination Improves CSF Contrast

**Fig. 4:**
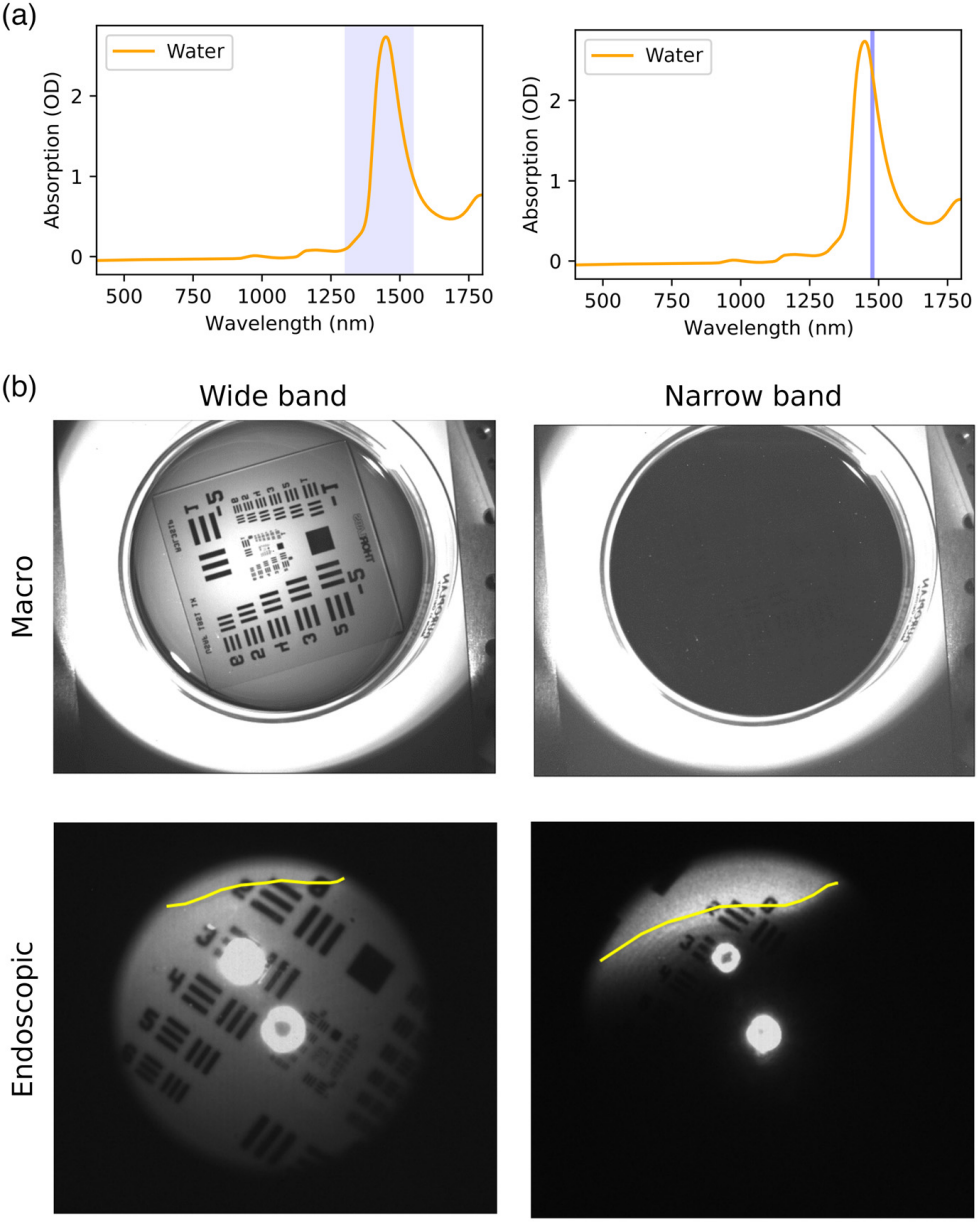
The effect of a narrower wavelength range on the absorption (and thus detection) of water. (a) The colored bands in the spectra show the wavelength range for the illumination of the images. (b) When narrowing the wavelength range the absorption of the water clearly increases, making the water appear darker and less translucent. This becomes apparent when viewing the test target with the different wavelength ranges, and the effect is present both in a macroimaging setup as well as in the endoscopic imaging setup. The yellow line on the endoscopic images shows the edge of the water.

Previous data had shown that droplets of water and thick films of water gave good contrast to surrounding tissue; thin films on the other hand were much harder to depict as they showed too little absorption to contrast clearly against the background. We aimed to improve this by moving the illumination window to a narrower range and focusing it more on the attenuation peak we had seen in the spectroscopic data [Fig. 4(a)]. The resulting images showed that narrowing the spectrum had a strong effect on the transparency of the water. Although it was clearly possible to depict the test target under the thin layer of water when using the broadband LED, this was no longer possible when adding the bandpass filter [Fig. 4(b)]. This increase in opacity when moving to the narrower illumination wavelength range meant that a trickle of water on top of tissue (chicken skin) could be seen much clearer than when using the 1450-nm wideband illumination (Fig. S1 in the Supplementary Material).

We adapted these findings to our endoscopic setup and exchanged the previously used broadband LED (M1450L4, Thorlabs) for a 1480-nm narrowband laser (L1480G1, Thorlabs). The laser allows for improved optical efficiency and provides additional power at the narrowband of 1480 nm, which we would lose with a broadband LED and filter setup. We analyzed the effect that had on the endoscopic image in a similar experiment to the one described above for the macrosetup. We again used a partially water-filled Petri dish laid on top of a test target. To be able to assess the effect the depth of the water had on its transparency, we slanted the Petri dish to create a gradient of water thickness. We added a very small amount of dish soap to the water to reduce the surface tension of the water and thus create a thinner film of water. The resulting images showed that the laser gave comparable images to those where the LED spectrum had been narrowed using a bandpass filter, and that the laser spectrum was sufficiently narrow for our purposes without requiring an additional bandpass filter. Although the test target was clearly visible throughout the entire image with the broadband illumination, it was only clearly visible for very thin films (≪1  mm) when using the narrowband illumination. With an increasing thickness of the film of water, it was no longer possible to perceive the test target [Fig. 4(b)].

**Fig. 5:**
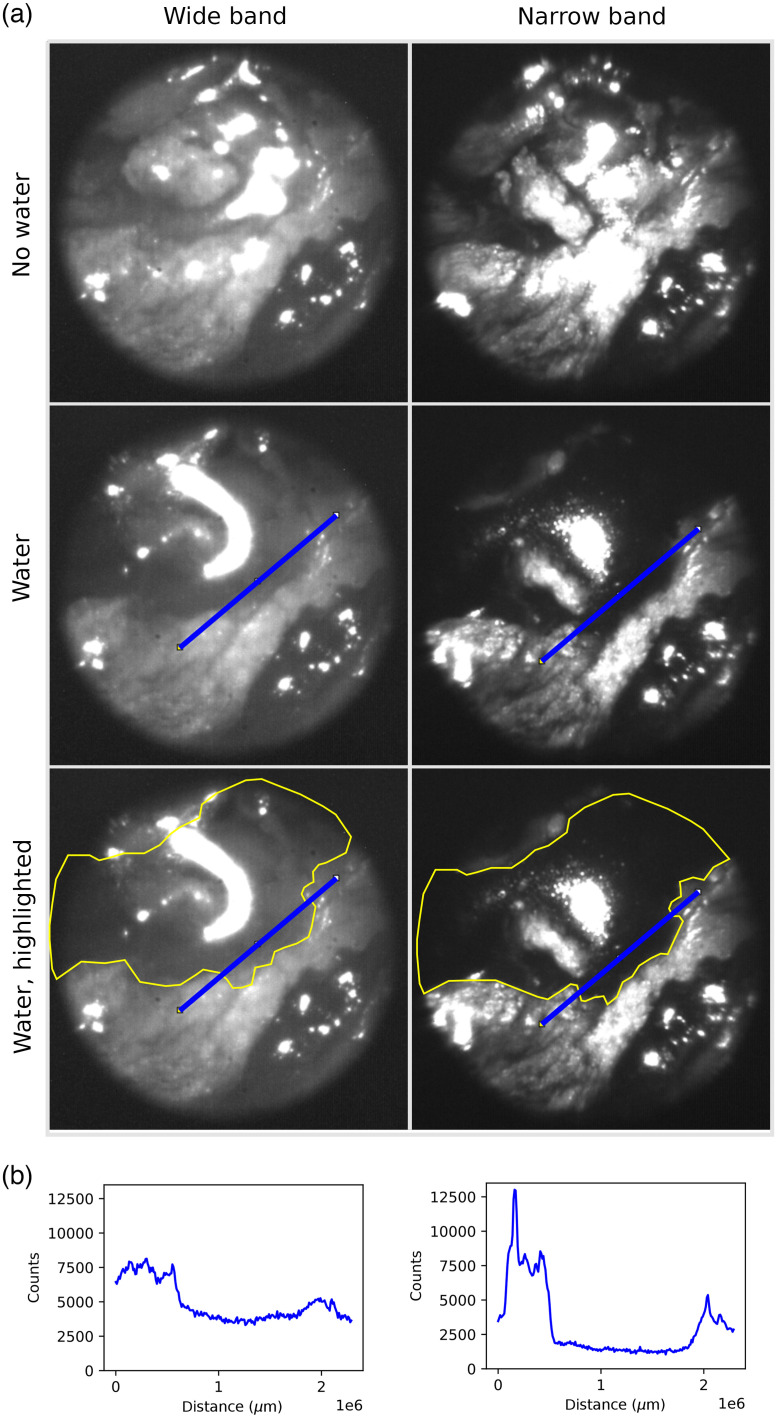
The effect of a narrower wavelength range when imaging tissue. (a) The narrowing of the wavelength range increases the overall contrast of tissue structures. It also makes it easier to pick out water on top of tissue, and it has an especially large effect on the edges and thinner areas of water. These images were taken without the use of polarizers. (b) The plot of the blue line profiles seen on the images above. The line profile cuts the edge of the water area. In line profiles over the water, we see that the narrowband image has higher contrast than the broadband image.

As a final test, we then imaged water on top of tissue using the endoscopic setup with the narrowband laser illumination. We used the same endoscope setup as had been previously used to image the Petri dish with water, only swapping the Petri dish out for a piece of chicken skin. We imaged the chicken skin without water and while running a droplet of water across it. The resulting images showed that narrowing the illumination helped to depict where water was present on the sample (Fig. 5). This result reflected the result we had seen when comparing the 1450-nm LED wideband illumination to the 1450 nm + 1480-12 bandpass filter narrowband illumination in the macrosetup. Narrowing the illumination spectrum around the absorption peak of water increases the contrast between tissue and water and thus the chances of being able to pick up smaller amounts of water against the surrounding tissue.

### 3.4 Reducing Reflections Through the Use of Polarizers

In the previous data, we observe that some areas of the images are overexposed, due to the presence of direct reflections of the illumination source of the sample. We aimed to reduce these bright reflections by adding polarizers to our endoscope design. One polarizer was positioned inside the video coupler (between endoscope and camera) and a second polarizer was attached to the tip of the endoscope. The polarizer at the tip of the endoscope needed to be custom cut to only cover the fiber ring of the endoscope and not the lens. The polarizers were oriented in such a way to each other as to bring the reflections to a minimum. Using this modified endoscope setup, we again imaged a piece of chicken skin, first without water and then while running water across it (Fig. S2 in the Supplementary Material). Comparing the images we obtained with and without polarizers, we can show that adding polarizers to the design gives us two positive effects: First, reducing the bright reflections, we are able to analyze more of the image accurately. Before, the regions displaying bright reflections could not be analyzed, as it was impossible to tell whether the tissue was causing the reflections, or whether the surface of the water was causing the reflections. Adding the polarizers reduced the size of overexposed areas on the images, thereby increasing the amount of information that could be obtained from one image. Second, reducing the bright reflections allowed us to increase the overall illumination; this in turn enhanced the contrast between tissue and water.

### 3.5 Comparison of Calculated and Measured Values

**Fig. 6:**
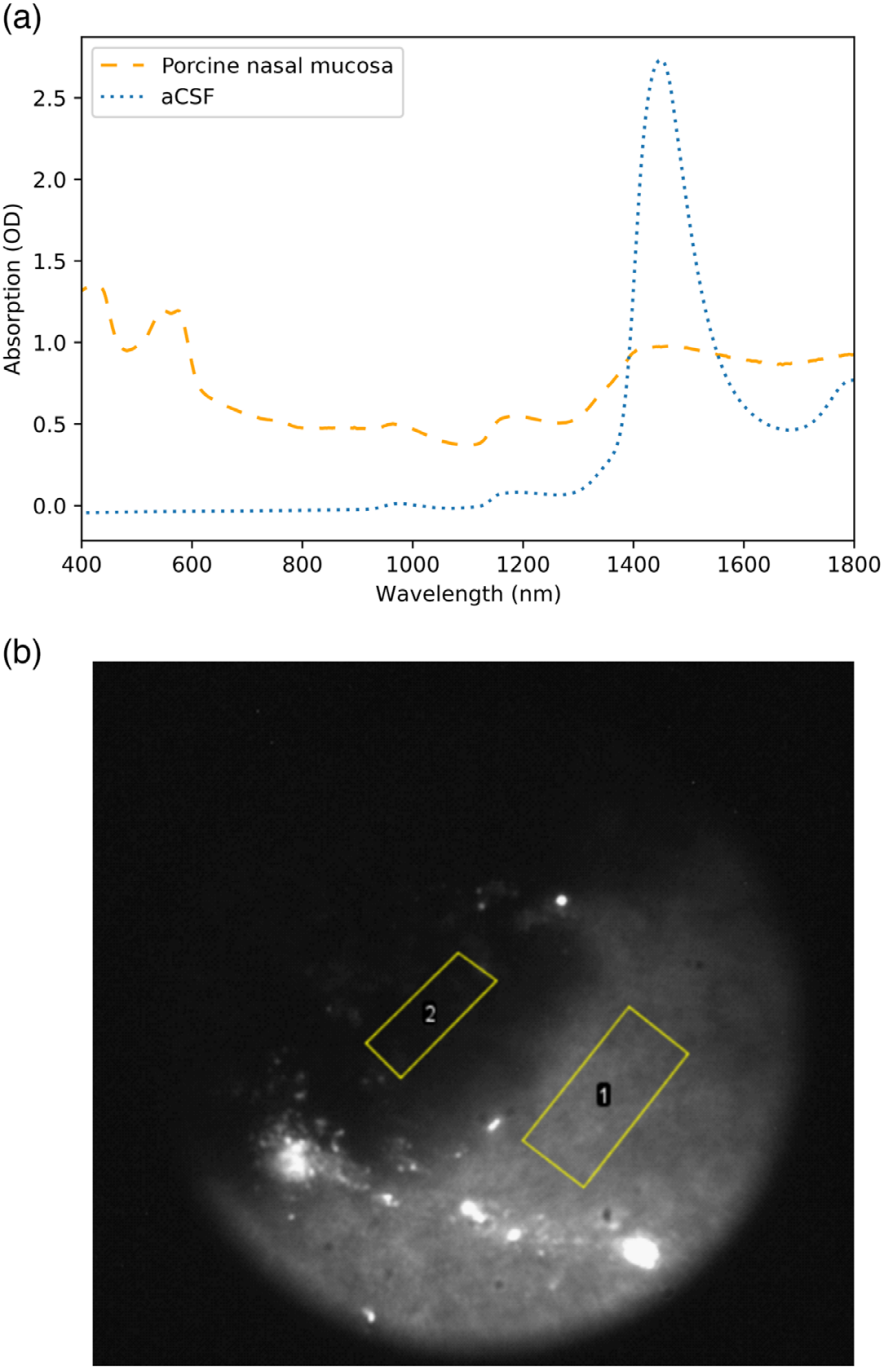
(a) Absorption spectrum of aCSF (path length of 2 mm) versus reflectance spectrum of a piece of porcine nasal mucosa (including underlying structures). From these data, we would expect to get roughly two and a half times as much signal from the nasal mucosa compared to a 1-mm film of aCSF. (b) Image of pig nasal mucosa with a pool of ca. 1-mm deep aCSF.

To check how well our improved SWIR endoscope performed we estimated the contrast, we would expect to see between porcine nasal tissue and aCSF. We compared a reflection spectrum of porcine nasal tissue to the transmission spectrum of aCSF. The absorption value for aCSF at 1480 nm was at around 2.5 OD for a path length of 2 mm. At this wavelength, the porcine nasal tissue had an OD of around 1 [Fig. 6(a)]. We would, therefore, expect to see 2.5× less signal from the aCSF compared to the tissue if we are looking at an aCSF depth of 1 mm.

We imaged a thin pool of aCSF on top of porcine nasal tissue using our endoscopic SWIR device. Region of interest (ROI) 1 shows porcine nasal tissue, and ROI 2 shows aCSF of roughly 1 mm of depth [Fig. 6(b)]. The ROIs have the following counts (see Table 1).

**Table 1:** Contrast between porcine nasal mucosa and aCSF.

	Calculated (absorption spectra)	Measured (reflection image)
aCSF	OD 2.5	11,664 counts
Porcine Nasal Mucosa	OD 1	28,828 counts
Ratio (reflection)	1:2.5	1:2.47

Our measured ratio lies very close to the calculated value, showing that the modifications we made to the endoscope have led to a very efficient imaging of fluid.

### 3.6 Imaging the Nasal Cavity

**Fig. 7:**
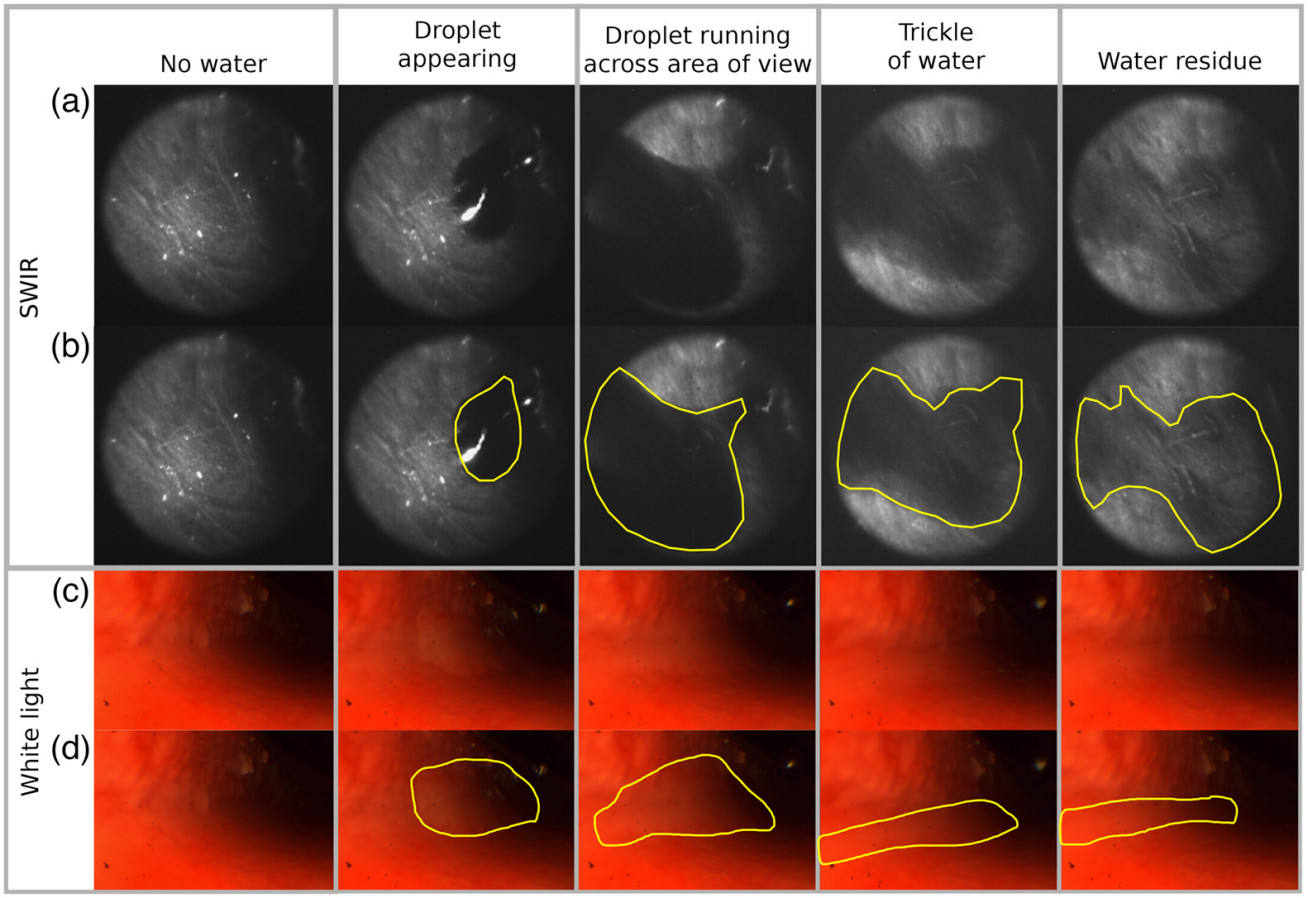
Images of the nasal cavity showing: no water, droplet appearing, droplet running across area of view, a trickle of water following in the path that the droplet left, and residue of water. Images from (a) and (b) the SWIR endoscope; (c) and (d) a white light endoscope. (b), (d) The water highlighted and otherwise identical to the images above them. With the revised endoscope design (including the polarizers and the narrow wavelength illumination), we significantly improved upon the quality of the data compared to our initial cadaver imaging. There are now fewer distracting reflections present, enabling us to clearly analyze almost the entire image and needing to omit very little areas in the image from analysis. Not only large droplets but also thin films and even small residues of water can be picked up against the tissue. In contrast, in the visible it is extremely difficult, if not almost impossible, to accurately say whether water is present or not. When viewing a video rather than still images, the differences between the imaging channels become even clearer (Videos 1 and 2). An issue in the SWIR imaging that needs to be addressed in further work is differentiating between shadows and water. In all images, it is not possible to accurately diagnose water in the areas that are not well illuminated. (Video 1, mp4, 10.8 MB [URL: https://doi.org/10.1117/1.JBO.28.9.094803.s1]; Video 2, mp4, 8 MB https://doi.org/10.1117/1.JBO.28.9.094803.s2).

For the final assessment of the improved SWIR endoscope design, we used a pig head that was halved along the saggital plane (food grade) and imaged the nasal cavity. The endoscope setup was identical to the setup described in Sec. 2.1. This setup combines the use of a narrowband illumination with the polarizers. Water was dripped into the nasal cavity near the skull base and trickled toward the tip of the nose. The nasal cavity was imaged without water present and while the water was trickling along it (Fig. 7). The same experiment was repeated using the white light endoscope.

Using the pig head, we were able to show that we were able to detect both droplets of water and thin films of water inside a nasal cavity. Even water residue left behind on the tissue could be detected, showing that we were able to greatly improve upon our initial endoscope design. In contrast, when using the white light endoscope, it was much harder to say whether water was present or not. The difference between the SWIR images and the white light images become even more evident when viewed as video (see Videos 1 and 2).

## 4 Discussion

CSF leaks can occur in an iatrogenic, traumatic, or idiopathic setting. If not identified in a timely manner, they can lead to complications ranging from headaches to meningitis. Current options for CSF leak diagnosis include the off-label use of intrathecal fluorescein and subsequent visualization with nasal endoscopy, cisternogram with intrathecal contrast, or testing for the CSF specific compound Beta-2 transferrin.^6^ Each method has its drawbacks. Both fluorescein and contrast require intrathecal delivery via a lumbar puncture (LP), which can cause significant discomfort and post-LP headache. Additionally, fluorescein injection has been associated with rare but serious side effects including paraparesis, numbness, and seizure.^14^ Moreover, the absence of fluorescein visualization can have a false-negative rate of as high as 26.2% in some studies (e.g., Ref. 3). Beta-2 transferrin, on the other hand, requires ample volume of rhinorrhea to be present and often has a 24- to 72-h delay for results.^15^ Thus a more accurate, immediate, and accessible means of intraoperative CSF leak detection is needed. We propose the use of a surgical system using an endoscope in the SWIR region for endonasal assessment of CSF leaks. This technology offers the opportunity to aid in the diagnosis of CSF leaks quickly, at the point of care, and with minimal morbidity.

Prior to developing an endoscopic system, we obtained spectra of CSF, aCSF, and water which were found to be identical. Because CSF is 99% water having a large absorption in the SWIR band around 1400 to 1500 nm, we are confident that this device has the potential to be an effective means of diagnosis. Human CSF can contain contaminants, such as proteins, metabolites, or cells of varying amounts, however, even in pathological states, these contaminants are only present in very small amounts and we still expect to clearly be able to depict CSF as fluid accumulation, as CSF will continue to be largely made up of water, and our device is focused on the absorption peak of water.

We have shown that it provides sufficient contrast between the aCSF (which appears as black since it readily absorbs SWIR light) and the surrounding tissue. Based on the absorption spectra, we customized an endoscope with those wavelengths characteristics into a form factor used on endoscopic sinus surgery. It was tested on a 3D-printed model simulating the nasal cavity and sinuses.

Testing our design on a cadaver showed that when using a broadband 1450-nm LED light source, we were not able to meet our criteria for CSF detection. The poor water absorption of thin layers of fluid showed diminished contrast and while illuminating the target area directly, there was a significant amount of reflections, which reduced our ability to accurately detect artificial CSF. Modifications on our illumination source included incorporation of a laser with a narrowband wavelength of 1480 nm to enhance contrast from water absorption. To decrease the reflection artifact, we incorporated polarizers that improved our detection accuracy. This was corroborated using porcine nasal cavity mucosa.

To be able to use the endoscope in a clinical setting, further modification will be needed. The size and weight of the camera are important (and potentially limiting) factors, as both can interfere with the handling and ease of use of the endoscope. However, the endoscope is compatible with any standard c-mount SWIR camera, and small and lightweight SWIR cameras are increasingly becoming commercially available. Cameras for this setup do not require extensive cooling to very low temperatures: we used one camera without cooling (Owl) and one camera with air cooling (Goldeye), and both were able to image at a frame rate suitable for real-time video feedback. For all endoscopic images, we were working at a frame rate between 20 and 100 fps (exposure times of 10 to 50 ms), which meant that dark noise due to little or no cooling was not a restricting factor for us. While high-end cooling is necessary for low light or low signal situations, when working with long exposure times, it is not of benefit for our device. On the contrary, a camera with high-end cooling would have more drawbacks than benefits if used with the endoscope, as the cooling adds size and weight to the camera, therefore, having significant negative effects on the usability of the endoscope as a hand-held device. An ideal camera for the endoscope is lightweight and small and capable of a frame rate suitable for real-time video feedback.

Another necessary modification is the mounting of the polarizer. While significantly improving image quality, the current polarizer setup has drawbacks on handling and ease of use of the endoscope. The polarizers would need to be revised and ideally manufactured directly into the endoscope in such a way that they stand up to the rigorous cleaning and disinfection needed for clinical use. Further, we would incorporate the polarizers into the video coupler in such a way that the camera can be freely rotated without impacting the polarizer orientation.

A further beneficial modification would be the incorporation of real-time dark and flatfield corrections, to give the user the best image quality possible.

Limitations of our current study include the inability to image both on the visible and SWIR which would be required for surgical procedures. Another limitation of our study is that our device has only been tested in cadavers and animal models which although valid testing models it does not represent the live scenario where blood and mucous secretions can be present. Moreover, currently used endoscopes and imaging systems for skull base surgery incorporate infrared imaging for ICG visualization to monitor perfusion of septal flaps.

Future work will aim to combine more than one imaging channel in the device for two reasons as follows.

1) We currently have no way of differentiating blood and CSF if we are only using the SWIR endoscope. By adding a second imaging channel with a wavelength that is absorbed by blood but not by CSF, we will be able to eliminate blood as a confounder. Ideally this channel would be a white light channel, as having both SWIR and white light combined in once device would bring significant benefits for the user, compared to needing two different devices to image either white light or SWIR. Should it not be possible to incorporate a white light channel, then a second SWIR channel would also be suitable for differentiating blood from other fluids.
2) At the moment, we see fluid as well as shadows/areas with poor illumination as dark areas in our images, sometimes making it difficult to differentiate fluid from areas with little illumination. Adding an imaging channel in which CSF does not have a significant amount of absorption could be used to calculate the difference between fluids and insufficient illumination, as only areas of insufficient illumination would appear dark in the second imaging channel.

One limitation of the device is that it cannot be used to diagnose CSF with certainty, as other watery fluids, such as interstitial fluid, blood, or mucus, will have a very similar absorption peak and thus also appear as dark areas when imaging. One method of eliminating blood as a confounder has already been explored above; however, interstitial fluid and mucus will be harder to differentiate from CSF. Mucus would not be expected to form droplets or trickles directly in the area of surgery and nowhere else, thus the location of the fluid accumulation can help to differentiate mucus from a potential CSF leak. Increased amounts of interstitial fluid are mostly associated with inflammation, meaning that during white light inspection one would see some swelling and this would be submucosal unlike a CSF leak. Our device cannot give certainty as to whether the fluid identified is CSF or some other type of fluid but it is a safer proposition than intrathecal fluorescein. It can, however, be used as an instrument to quickly and routinely check for abnormally large amounts of fluid accumulation and is very sensitive in picking up abnormalities of fluid presence. This knowledge can then be combined with other tools that the doctor has on hand to diagnose CSF leaks quicker and earlier than currently possible and to enable routine screenings of patients at risk of developing a CSF leak.

Our future work will look to incorporate the ability to image both on the SWIR and the visible and also to incorporate fluorescence imaging while maintaining a weight and form factor that allows use in the operating room and can tolerate the rigors of surgical sterilization procedures.

## Code, Data, and Materials Availability

The data was made publically available at https://www.ebi.ac.uk/biostudies/bioimages/studies/S-BIAD658.
