# Supplementary material for: Development of a shortwave infrared sinuscope for the detection of cerebrospinal fluid leaks
Source: J Biomed Opt. 2023 May 12;28(9):094803. doi: 10.1117/1.JBO.28.9.094803 (PMC10181794; doi:10.1117/1.JBO.28.9.094803)
Supplement: Supplementary file 1 [file JBO_028_094803_SD001.pdf]

Supplementary Material for “Development of a shortwave infrared (SWIR) sinuscope for the detection of cerebrospinal fluid (CSF) leaks.”

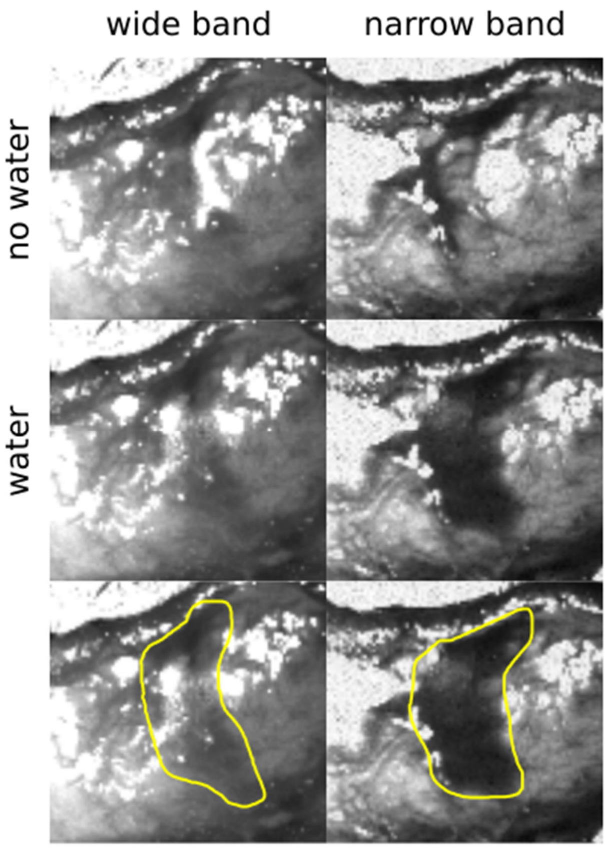

Fig. S1 Piece of chicken skin. Left column is with a 1450nm LED, no filters. Right column is with a 1450nm LED and 1480-12 bandpass filter. The top row is without water present, the middle and bottom row are with a film of water present (water highlighted in the bottom row). With the bandpass filter, the contrast between the chicken skin and the water increases, compared to the LED without the bandpass filter.

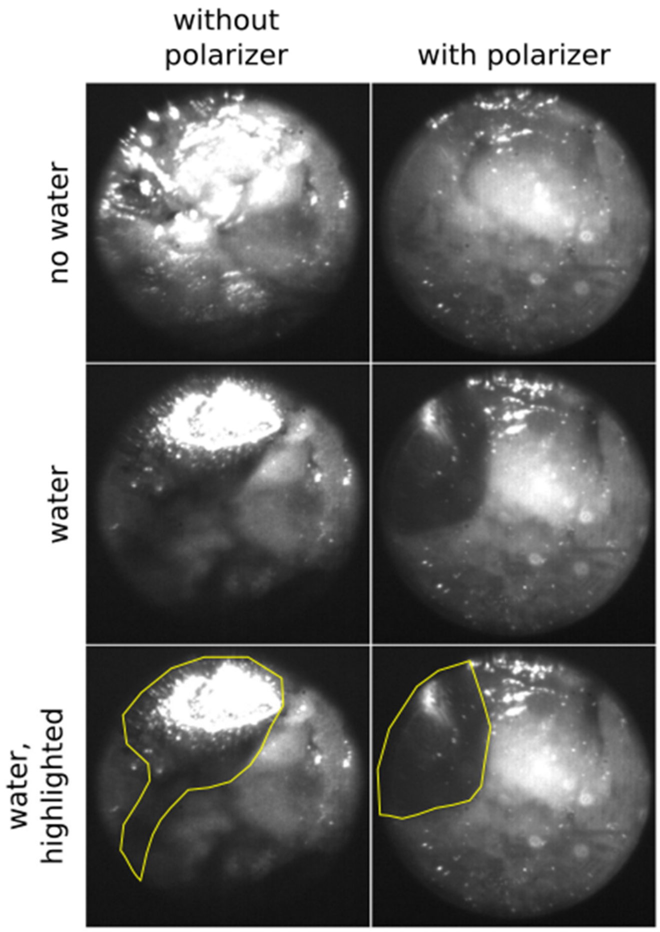

Fig. S2 Comparison of imaging with and without the use of polarizers. The use of polarizers significantly reduces the overexposed areas in the image. This means that less of the areas of the image need to be omitted when analyzing and the presence of water in the image can be labeled with more certainty. The images were taken using the narrow band 1480nm laser for illumination.
